# Protecting the aging mind: how cognitive reserve and lifestyle factors relate to executive functions and long-term memory

**DOI:** 10.1007/s40520-026-03362-y

**Published:** 2026-03-26

**Authors:** Giulia Scotti, Greta Caprara, Chiara Penserini, Lucilla Titta, Valeria Cuccarini, Manuela Berlingeri, Cristina Rosazza

**Affiliations:** 1 Department of Humanities (DISTUM), Università degli Studi di Urbino Carlo Bo, Urbino, Italy; 2 Department of Experimental Oncology, IEO, European Institute of Oncology IRCCS, Milan, Italy; 3 Department of Neuroradiology, Fondazione IRCCS Istituto Neurologico Carlo Besta, Milan, Italy

**Keywords:** Aging and active aging, Cognitive reserve, Physical activity, Healthy diet, Sleep, Executive functions

## Abstract

**Background:**

With an aging population, identifying factors that support brain health and mitigate cognitive decline has become increasingly important. Among these, modifiable lifestyle factors such as cognitive reserve, physical activity, diet and sleep play a key role in healthy aging. However, the specific contribution of each factor and their role in the different cognitive domains remain unclear.

**Aims & methods:**

We investigated the role of four lifestyle factors -cognitive reserve, lifelong physical activity, dietary habits, and sleep duration- on executive functions and long-term memory in a sample of 204 cognitively healthy older adults. In this cross-sectional study, participants were assessed remotely through individual online sessions, including neuropsychological testing and standardized questionnaires. Confounding variables (sex, smoking, alcohol consumption, and number of chronic conditions) were controlled for in additive and moderation models, implemented through hierarchical general linear models.

**Results:**

The results revealed differential effects across cognitive domains: lifestyle factors predicted executive functioning, while their association with long-term memory was limited. Individuals who adopted all four lifestyle behaviors exhibited executive function performance higher than 65% of the sample. Significant interactions emerged in the executive domain, indicating that the benefits of physical activity and a healthy diet were stronger among individuals with lower cognitive reserve, suggesting a compensatory rather than enhancing effect. Among all predictors, cognitive reserve explained the largest proportion of variance, followed by physical activity, diet, and sleep.

**Discussion & conclusions:**

Our findings emphasize the cumulative and interactive role of lifestyle factors in supporting cognition in aging, particularly executive functions, with differential associations between cognitive reserve and lifestyle factors.

**Supplementary Information:**

The online version contains supplementary material available at 10.1007/s40520-026-03362-y.

## Introduction

Aging is characterized by cerebral (e.g. volume reduction and atrophy), neurofunctional (e.g. loss of specialization and compensatory mechanisms) and cognitive changes, leading to considerable variability in cognitive performance among older adults [1, 2]. This variability may partly depend on biological factors such as genetics and sex differences, and environmental or lifestyle factors, such as cognitive engagement, physical activity, dietary habits, alcohol consumption and sleep [2, 3]. These two categories of factors significantly interact, shaping the age-related cognitive changes across lifespan.

With aging, cognitive performance declines across various domains, but not uniformly or at the same rate [4]. Certain abilities such as vocabulary tend to remain relatively stable throughout life [2], while others such as processing speed are more sensitive to aging and exhibit a steady decline over time [4, 5]. In particular, executive functions including working memory, cognitive flexibility (shifting) and inhibitory control begin to decline from 20’s, remain relatively stable during midlife and tend to deteriorate more markedly after the age of 60 [4, 6]. Working memory shows a gradual decline over time [2, 7] and a similar pattern is observed for cognitive flexibility, with a more pronounced reduction observed after age 65 [8]. Inhibitory control also declines with age, as typically assessed using the Stroop test which reveals slower response times and greater difficulty inhibiting automatic responses from age 65 [9]. Memory, on the other hand, follows different trajectories depending on its components. Episodic memory remains relatively stable until the age 60–65 according to longitudinal studies; then, after age 70 a significant change is observed [2]. In contrast, semantic memory increases between 35 and 55 years, then stabilizes, with only a slight decline after age 65 [2]. This decline is considerably slower than that of episodic memory, emphasizing the importance of lifelong learning [10].

Despite these declines, the brain retains a remarkable capacity to adapt to both environmental changes and internal states by recruiting compensatory processes [11, 12] and building cognitive reserve [2, 4, 13]. With an aging population, there is a growing interest in identifying key factors that support brain health and slow cognitive decline [14]. Among these, modifiable lifestyle factors such as cognitive engagement, physical activity, dietary habits, and sleep play a crucial role.

Lifelong cognitive engagement is known to contribute to cognitive reserve, promoting neural plasticity by strengthening neural connections, preserving brain volume, and fostering compensatory mechanisms [15, 16]. Regular cognitive challenges have been linked to prolonged cognitive function and a lower risk of dementia [17, 18].

Exercise enhances cerebral blood flow, improves oxygen and nutrient delivery while stimulating Brain-Derived Neurotrophic Factor (BDNF), which supports neurogenesis and synaptic plasticity [19, 20]. It also reduces inflammation and oxidative stress, providing neuroprotective effects and slowing neurodegeneration. On a cognitive level, regular movement enhances executive functions and contributes to better memory and mood by reducing anxiety, depression, and stress [21, 22].

A healthy diet supports cognitive function by reducing inflammation and oxidative stress [23–26]. Adherence to dietary patterns like the Mediterranean Diet and Mediterranean-DASH Intervention for Neurodegenerative Delay (MIND) diets is associated with slower cognitive decline and a reduced risk of dementia [27–30].

Finally, sleep is a critical factor in cognitive health, supporting memory consolidation, emotional regulation, and neuroprotection [31–33]. Both quality and duration of sleep influence brain function, and disruptions are linked to cognitive impairment [33, 34].

In the literature, these factors are often analyzed together (e.g., diet, exercise and other variables, as in Ngandu et al. [35]), combined into an index (e.g., a total lifestyle index, as in Anastasiou et al. [26]), or grouped by the number of lifestyle factors (e.g., 0–1, 2, 3–5, as in Weng et al. [36]). For instance, in a 10-year longitudinal study of more than 29,000 older adults, six lifestyle factors including cognitive activity, regular physical activity and healthy diet were examined together in a single aggregated score [37]. Participants with more healthy lifestyle factors exhibited slower memory decline than those with fewer or none. This methodological design with multiple lifestyle factors analyzed together closely reflects real-life conditions, but makes it difficult to disentangle the individual contribution of each factor and to assess their relative importance. Moreover, the literature shows that these variables are generally associated with cognitive benefits [14], particularly in executive functions [35, 38], and also in memory [37, 39]. However, many studies have relied on global cognitive scores, which may mask domain-specific effects. Further evidence is therefore needed to clarify the differential relationships between lifestyle factors and specific cognitive domains.

In this cross-sectional study, we focused on four lifestyle factors (cognitive reserve, lifelong physical activity, dietary habits, and sleep duration) and investigated their association with cognitive performances in two domains of interest (executive functions and long-term memory) in a sample of healthy older adults.

Prior evidence consistently supports the relevance of these factors on cognition, but their relative contribution remains debated; therefore, we adopted a data-driven approach in which the strength of the relationship (i.e., R^2^) between every single predictor and each domain of interest was explored using simple linear regressions. This approach allowed us to determine the entering order of the predictors into a hierarchical structure, minimizing a-priori assumptions. Accordingly, General Linear Models (GLM) were applied to estimate the contribution of each factor and to test interaction effects on executive functions and episodic long-term memory.

## Materials and methods

### Participants

A total of 207 participants were recruited. After excluding three outliers, the final sample consisted of 204 cognitively healthy older adults (aged 65–100), tested between April 2023 and April 2024. Inclusion criteria were: a) age over 65 years; b) native Italian speaker; c) absence of any previous or current neurological disorders such as stroke, epilepsy or head trauma; d) no visual impairments (e.g., maculopathy); e) no history of previous or current chemotherapy treatment; f) no prior experience with the neuropsychological tests used in the study; g) normal performance on Mini-Mental State Examination (MMSE [47]; adjusted score ≥ 26.02); and h) having access to an electronic device connected to the Internet.

Participants were recruited through the authors’ network, local associations (e.g., Confartigianato Imprese di Ancona-Pesaro e Urbino, Memorabilia, Universalia3, Accademia Europea di dinamica mentale), social media and leafleting. The study was approved by the Ethical Committee of the University of Urbino Carlo Bo, Italy (N 31/2020) and was conducted in accordance with the ethical standards of the Declaration of Helsinki. Written informed consent was obtained from all subjects. The study was funded by Fondazione Asino.

### Assessment

Participants underwent a semi-structured interview and a neuropsychological assessment conducted remotely through individual online video meetings, in accordance with the recommendations on remote testing reported by Rizzi and colleagues [40].

Sociodemographic and general medical information were collected, including region of residence (north, center, south of Italy), smoking status (yes, no, ex-smoker), alcohol consumption (never, 1–2 month, 1–2 week, 5–7 week), presence of chronic conditions (vascular, oncologic and diabetes), living arrangement (alone or with others), body mass index (BMI, Kg/m^2^). Descriptive characteristics are reported in Table 1.

**Table 1:** Demographic data of the sample (N = 204). In the first column, mean (SD) and percentages are reported. * Refers to the score excluding the wine item.

Main characteristics	Total	range
Age, years	72.9 ± 6.65	65—100
Sex, %F	59%	
Education, years	12.3 ± 4.58	3—30
Region, %north/center/south	58% 38% 4%	
Smoking, %no/ex-smoker/yes/	44% 51% 4%	
Alcohol,%never/ 1-2 month / 1-2 week / 5-7 week	30% 16% 18% 36%	
Chronic conditions (0–3 pathologies), %0/1/2/3	59% 32% 8% 0.5%	
Living arrangement, %alone	29%	
BMI, Kg/m2	25.2 ± 4.22	18.4—41.9
BIS-15	25 ± 5.57	15—42
HADS-A	2.80 ± 2.31	0—12
HADS-D	1 ± 1.37	0—9
CRIq	119 ± 18.9	71—178
MRIq	43.7 ± 9.3	9.72—66.40
MRIq—Sect.	22.6 ± 20.1	0—79
Sleep, hours per night	6.47 ± 1.22	3—9.5
Med-diet *	7.82 ± 1.69	4—12
MIND diet *	8.95 ± 1.27	5.5—11.5

Barratt Impulsiveness Scale (BIS-15); Hospital Anxiety and Depression Scale (HADS); HADS-A = Hospital Anxiety and Depression Scale–Anxiety subscale; HADS-D = Hospital Anxiety and Depression Scale–Depression subscale; Cognitive Reserve Index questionnaire (CRIq); Motor Reserve Index questionnaire (MRIq); Mediterranean Diet questionnaire (Med-diet); Mediterranean-DASH Intervention for Neurodegenerative Delay questionnaire (MIND diet).

The lifestyle factors of interest were measured through:

- The *Cognitive Reserve Index questionnaire* (CRIq; [41]) to assess the amount of cognitive reserve acquired during a lifetime. The CRIq consists of 20 items grouped into three sections: education (i.e., years of formal and informal education), working activity (i.e., years and level of professional occupation), and leisure time (i.e., years of frequent engagement in various activities) during the lifetime starting from 18 years old. Each activity is scored according to its frequency and the years of practice; the final score is computed as the average of the scores obtained for the three sections.
- The *Motor Reserve Index questionnaire* (MRIq) to quantify the physical activity practiced throughout life [42]. Here we focused on Section IV’s score corresponding to light and vigorous physical exercise performed during the lifetime starting from 18 years old. The score is computed according to the rules reported by Pucci and colleagues [42].
- The *Mediterranean Diet questionnaire* (Med-diet) to assess adherence to the traditional Mediterranean diet [43]. The Med-diet questionnaire consists of 14 items related to the consumption of the following dietary components: olive oil, vegetables, fresh fruit, red meat, butter, sugary drinks, wine, legumes, fish, sweets, nuts, white meat, and use of “sofrito”, an aromatic mix of onions, carrots and celery cooked in olive oil. The questionnaire was adapted to the Italian dietary habits by making minor revisions (e.g., portion sizes corresponded to those referenced for the Italian population) as defined by the Italian Society of Human Nutrition (SINU – LARN IV Revision [44]). Each component is assigned a score of 0 or 1 based on its daily or weekly consumption frequency. The total Med-diet score was calculated by summing the item responses, excluding wine consumption, as it was deemed non-protective [45, 46]. Total score ranged from 0 (lowest) to 13 (highest) diet adherence. The questionnaire was administered through Google Forms.
- The *Mediterranean-DASH Intervention for Neurodegenerative Delay questionnaire* (MIND) to assess the adherence to the MIND diet [28, 29]. The MIND questionnaire consists of 15 items related to dietary components: 10 favorable for brain health (leafy green vegetables, other types of vegetables, nuts, berries, legumes, whole grains, fish, poultry, olive oil, and wine) and 5 unfavorable (red and processed meat, butter/margarine, cheese, sweets, and fried/fast food). The questionnaire was adapted to the Italian dietary habits by making minor revisions as defined by SINU LARN IV Revision [44]. Each component is assigned a score of 0, 0.5, or 1 based on its daily or weekly consumption frequency. The total MIND score was calculated by summing the item responses, excluding wine consumption, for the same above reported reasons; therefore, the total score ranged from 0 (lowest) to 14 (highest) diet adherence. The questionnaire was administered through Google Forms.
- The number of hours slept during a typical night.

Finally, the participants underwent an online neuropsychological testing including: i) the Mini-Mental State Examination (MMSE [47]) to assess global cognitive functioning; ii) the Phonemic fluency (letters ‘‘A’’, ‘‘F’’ and ‘‘S’), Semantic fluency (categories “colours’’, ‘‘animals’’ and ‘‘fruits’’) and Alternating fluency (trial 1 letter ‘‘A’’ and ‘‘Colours’’; trial 2 letter ‘‘F’’ and ‘‘Animals’’; trial 3 letter ‘‘S’’ and ‘‘Fruits’’) [48], the shortened version of the Stroop test [49], the Raven’s Progressive Matrices (for non-verbal abstract reasoning) [50], the Alternation Subtest of the Edinburgh Cognitive and Behavioral ALS Screen (ECAS; [51] to assess executive functions); iii) the Digit Span Forward and Backward tests to assess short-term/working memory [52]; iv) the Free and Cued Selective Reminding Test to assess long-term memory (FCSRT; [53]).

At the end of the neuropsychological tests battery, participants were orally administered two self-report questionnaires: the short Italian version of the *Barratt Impulsiveness Scale* (BIS-15; [54]) and the *Hospital Anxiety and Depression Scale* (HADS) which includes two subscales: HADS-A (anxiety) and HADS-D (depression) [55, 56].

Visual stimuli were presented via screen sharing. For the MMSE time orientation item, participants were asked to close their eyes to respond; for the writing and copying items they completed the tasks on a blank paper and showed their responses to the examiner via webcam, allowing a screenshot to be taken.

The questionnaires and neuropsychological tests were administered in the same order (see Supplementary Table 1 for more details); video calls did not exceed 90 min.

### Statistical analyses

First, descriptive statistics were computed for each lifestyle and neuropsychological measure. Outliers were identified using the boxplot method, which is based on the interquartile range (IQR). Three outliers were identified and removed from the dataset (one for Med-diet and MIND questionnaires, one for MRIq and another one for a motor test); therefore, the final sample included 204 participants.

Second, the correlation matrix between neuropsychological measures (except for the MMSE which served as a screening test) was explored and tested by means of the Bartlett’s test. Consequently, a Principal Component Analysis (PCA) was run to group neuropsychological measures into higher order cognitive dimensions. Only components with an eigenvalue ≥ 1 were extracted and rotated with an Oblimin Rotation with Kaiser link (see Costello & Osborne [57] for a review) as, by definition, the neuropsychological tests included in our battery were highly correlated. The identification of the higher order cognitive domains represented by each component was based on the exploration of the relative loadings from each observed variable. Finally, for each participant, we computed the component scores using the regression-based method. These were used as dependent variables for subsequent analyses.

To examine the relationship between the cognitive components identified with the PCA and our lifestyle factors of interest (namely, cognitive reserve, motor reserve, dietary habit and sleep duration), a series of General Linear Models (GLM) were performed. To test whether the adoption of multiple lifestyle factors could significantly predict cognitive performance in the two domains, the variable ‘number of positive lifestyle factors’ was created for each participant. Specifically, participants were assigned a score of 1 (otherwise 0) for each of the following: CRIq above 115 [41], Mediterranean diet score ≥ 8 [63], and sleep duration ≥ 7 h per night [62]. For physical activity (MRIq), a data-driven approach was used by splitting the sample at the 25th percentile of the empirical distribution: participants scoring at or above this threshold were assigned 1, others 0. The resulting values (0 or 1 for each factor) were then summed to obtain the variable ‘number of positive lifestyle factors’ (range 0–4). This variable was entered into a linear regression analysis to assess the cumulative benefits of adopting multiple healthy behaviors across the adult lifespan, together with four confounding variables: sex (2 levels), smoking (3 levels), alcohol consumption (4 levels) and presence of chronic conditions (4 levels).

Finally, to explore possible interactions between our lifestyle factors of interest, a series of hierarchical GLM were performed. The entering order of the four lifestyle factors was based on the strength of the relationship (i.e., R^2^) between each single predictor and each domain of interest using simple linear regressions; the four confounding variables were always included. The multivariate GLM with the best fitting level was selected according to the Akaike Information Criterion (AIC). The analyses were performed using Jamovi version 2.5.7 software [58].

## Results

### Sample description

Participants completed the remote tasks without difficulty or caregiver support. No issues with internet connection or device functionality were reported.

As reported in Table 1, among the 204 healthy subjects, the mean age was 72.9 years (SD = 6.65); 59% were women and the educational level was medium (mean years of formal education = 12.3, SD = 4.58). Concerning the confounding variables, 51% were former smokers, 44% never smoked, and 4% were current smokers. Regarding health, 59% had no chronic conditions, 32% had one, 8% had two, and 0.5% had three. Concerning alcohol consumption, about one-third of participants reported no use (30%), another third reported frequent use (5–7 times per week; 36%) and the rest reported low-frequency consumption (34%). The sample was just above the normal weight range, with a mean BMI of 25.2 (SD = 4.2) [59].

Concerning the lifestyle variables of interest, CRIq scores were moderate to high (M = 119, SD = 18.9), while overall MRIq scores ranged from 9.72 to 66.40, a distribution comparable to that reported in Pucci et al. [42], with a mean of 22.6 for section IV. Scores on the Med-diet and MIND indicated moderate adherence to healthy dietary habits, with a mean of 7.82 (SD = 1.69; median = 8, excluding the wine item) for the Med-diet and a mean of 8.95 (SD = 1.27; median = 9, excluding the wine item) for the MIND, in line with other studies [28, 60]. The two scores were highly correlated (ρ = 0.42, *p* < 0.001). We chose to focus on the Med-diet, as some MIND items were generally uncommon or rarely consumed by the older population, resulting in very low scores (e.g., fast food, whole grains and berries which are seldom consumed outside the summer season). Finally, the number of sleep hours per night was between 3—9.5 (median = 7; mean = 6.47; SD = 1.22) with the majority of participants (34%) sleeping 7–8 h, 27% sleeping 6–7 h, 23% sleeping less than 6 h, and 15% sleeping more than 8 h, a pattern resembling the typical variability observed in aging [34, 61, 62].

### Neuropsychological tests and Principal Component Analysis (PCA) results

The overall performance of neuropsychological tests is reported in Table 2. None of the participants obtained an adjusted MMSE score below the cut-off [47].

**Table 2:** Performance of the sample (n = 204) on the neuropsychological tests

Test	Mean ± SD	range
MMSE	28.8 ± 1.14	25—30
Digit Span Forward	5.47 ± .91	3—9
Digit Span Backward	4.23 ± 1.06	2—8
Free and Cued (FCSRT) -IFR	28.8 ± 3.98	19—36
Free and Cued (FCSRT) -DFR	10.2 ± 1.45	7—12
Phonemic Fluency	33 ± 11.2	9—60
Semantic Fluency	45 ± 9.98	20—73
Alternate Fluency	30.6 ± 12.7	5—62
Stroop Test	24 ± 14.1	3—91.5
ECAS 12	10.2 ± 3.25	0—12
Raven’s Progressive Matrices	33.1 ± 7.1	14—45

Mean raw scores (and SD) are reported. Abbreviations: Mini-Mental State Examination (MMSE); Free and Cued Selective Reminding Test- Immediate Free Recall (FCRST- IFR); Free and Cued Selective Reminding Test- Delayed Free Recall (FCRST-DFR); Alternation Subtest of the Edinburgh Cognitive and Behavioral ALS Screen (ECAS 12).

A normal performance was observed on short-term and long-term memory tests, consistent with previous studies on healthy aging samples ([52, 63]). Scores within normal ranges were also observed for executive functions, including semantic and phonemic fluency, Stroop and Raven [63] [64] [65].

Using the PCA and the eigenvalue method, we identified two components: the first had an eigenvalue of 4.814 and the second had an eigenvalue of 1.181 (see also the scree-plot in Supplementary Fig. 1). Only the variables with loadings ≥|0.3| were considered. The two factors accounted for 60% of the variance (42.7% and 17.3%, respectively) and were labeled as follows: Factor 1 -Executive functions (EF), characterized by high loadings from tasks involving reasoning, WM and executive functions; Factor 2 -long-term memory, characterized by high loadings from the Free and Cued Task (Table 3). We computed the factors’ scores using a classic regression approach to obtain two new variables.

**Table 3:** Results of the Principal Component Analysis (PCA)

Test	Executive functions (EF)	Long-term memory
Free and Cued (FCSRT) -IFR		.887
Free and Cued (FCSRT) -DFR		.859
Raven’s Progressive Matrices	.824	
Digit Span Forward	.714	
Digit Span Backward	.694	
Phonemic Fluency	.693	
Semantic Fluency	.651	
Alternate Fluency	.843	
Stroop Test	-.614	
ECAS 12	.748	

MMSE, Mini-Mental State Examination. Abbreviations: Free and Cued Selective Reminding Test- Immediate Free Recall (FCRST- IFR); Free and Cued Selective Reminding Test- Delayed Free Recall (FCRST-DFR); Alternation Subtest of the Edinburgh Cognitive and Behavioral ALS Screen (ECAS 12).

### General linear models on factors extracted from PCA

The two variables extracted from the PCA (i.e., EF and long-term memory) were used as dependent variables in linear regression models: first in simple regression analyses, then in a series of hierarchical regression analyses. Confounding variables (i.e. sex, smoking, alcohol consumption and chronic conditions) were always included.

#### Study of executive functions (EF)

**Fig. 1:**
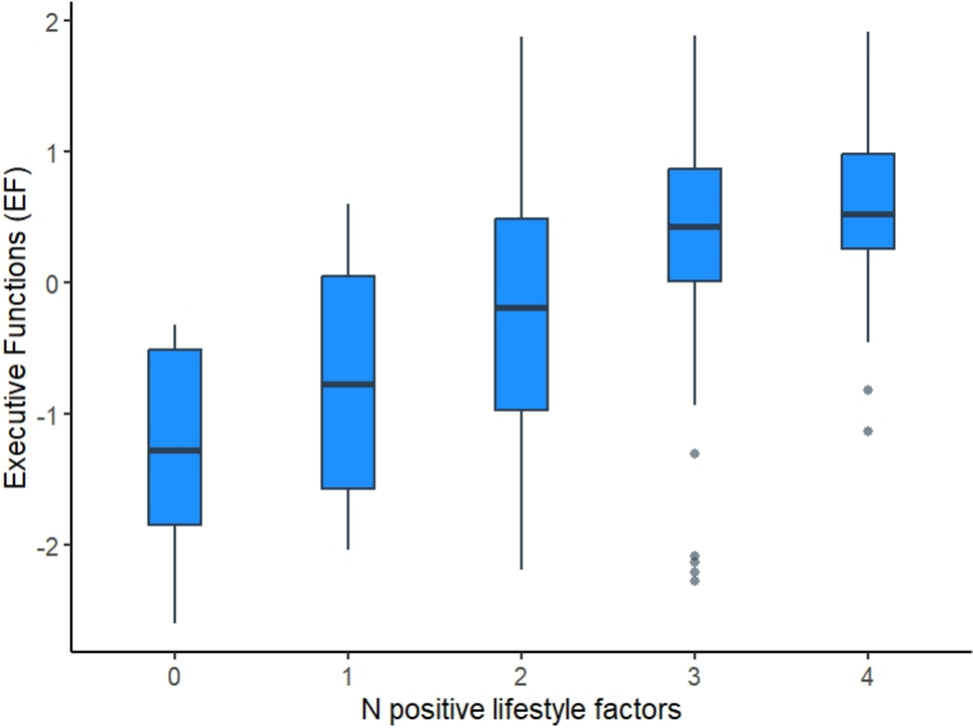
The number of lifestyle factors adopted (CRIq, MRIq, sleep, and Mediterranean diet) shows a significant linear association with executive functions (EF): participants adopting more healthy behaviors show progressively higher EF scores. Those with all four factors show an expected EF score of 0.46, corresponding to a performance of about 65% of the sample.

The regression analysis of the variable ‘number of positive lifestyle factors’ (i.e., CRIq, MRIq, sleep, and Mediterranean diet) on EF showed a clear linear trend: for each additional healthy behavior adopted, EF scores increased by approximately 0.46 points (Table 4 and Fig. 1). Participants who adopted all four healthy behaviors reached an expected EF score of 0.46, corresponding to a performance about 65% of the sample. The model explained 30% of the variance (Table 4). This effect suggests that each lifestyle factor contributes independently and cumulatively to better cognitive functioning.

**Table 4:** Results of the linear regression analyses on the number of positive lifestyle factors for both Executive Functions (EF) and Long-term Memory

	**R**^**2**^	**Adjusted R**^**2**^	***F***_**df**_	***p***	**Beta**	**Estimate**	**t**	***p***
*Executive Functions (EF)*								
N of positive lifestyle factors	.29	.25	7.90 (10,193)	< .001	.50	0.46	7.86	< .001
*Long-term Memory*								
N of positive lifestyle factors	.13	.089	2.98 (10, 193)	.002	.32	0.29	4.54	< .001

The statistics reported in this table are derived from a GLM including both the confounding variables and the variable of interest i.e., the number of positive lifestyle factors. As a consequence, the Beta values and the Estimates refer to effect of the number of positive lifestyle factors.

To explore the presence of potential interaction effects beyond the additive contributions of individual lifestyle factors, hierarchical regressions were performed with predictors entered according to their explanatory power, while controlling for confounding variables. The results are reported in Table 5.

**Table 5:** Results of the hierarchical regression analyses on the Executive Functions (EF) scores extracted from PCA.

	**R**^**2**^	**Adjusted R**^**2**^	**AIC**	***F***	***p***	**Beta**	**t**	***p***
MODEL 1	.42	.39	491	13.97 (10,193)	< .001			
CRIq						.62	10.9	< .001
MODEL 2	.44	.40	489	12.26 (12,191)	< .001			
CRIq						.58	8.72	< .001
MRIq						.10	2.17	< .05
MRIq*CRIq						-.12	-2.03	< .05
MODEL 3	.45	.41	487	11.81 (13,190)	< .001			
CRIq						.58	8.63	< .001
MRIq						.09	1.96	.051
MRIq*CRIq						-.11	-1.86	.065
sleep						.11	2.01	< .05
MODEL 4	.45	.41	489	10.92 (14,189)	< .001			
CRIq						.58	2.06	< .05
MRIq						.09	1.99	< .05
MRIq*CRIq						-.11	-1.89	.061
sleep						.12	-.019	.985
sleep*CRIq						.02	.35	.726
**MODEL 5**	**.47**	**.42**	**486**	**10.16 (16,187)**	** < .001**			
CRIq						.58	3.16	** < .005**
MRIq						.08	2.03	** < .05**
MRIq*CRIq						-.11	-1.96	**.052**
sleep						.12	-0.05	.963
sleep*CRIq						.02	.39	.697
Med-diet						-.02	2.22	** < 0.05**
Med-diet*CRIq						-.14	-2.37	** < 0.05**
MODEL 6	.47	.42	488	9.59 (17,186)	< .001			
CRIq						.58	3.15	< .005
MRIq						.08	1.9	.058
MRIq*CRIq						-.11	-2.12	.036
sleep						.11	.16	.876
sleep*CRIq						.02	.09	.932
Med-diet						-.03	2.35	.02
Med-diet*CRIq						-.13	-2.5	.013
Med-diet*CRIq*MRIq*sleep						-.004	.82	.416

Cognitive Reserve Index questionnaire (CRIq); Motor Reserve Index questionnaire (MRIq); Mediterranean Diet questionnaire (Med-diet)

**Fig. 2:**
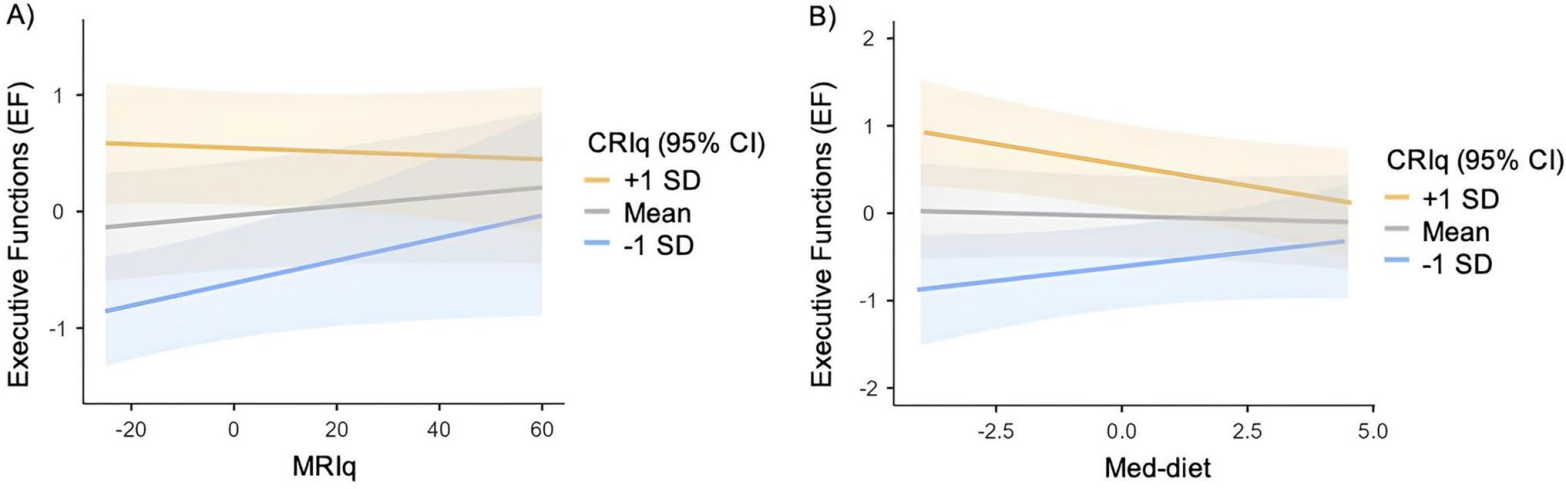
Two significant interactions in the regression analysis of executive functions (EF). Panel A) MRIq*CRIq interaction (β = -0.11, p = .052): the higher the physical activity questionnaire score, the higher the EF score in individuals with lower cognitive reserve (CRIq). Panel B) the Med-diet*CRIq interaction (β = -0.14, p < .05): the healthier the diet questionnaire score, the higher the EF score in individuals with lower CRIq.

Model 1 included the CRIq as predictor (R^2^ = 0.39, *p* < 0.001 in the single regression); in Model 2 we added MRIq (R^2^ = 0.072, *p* < 0.001 in the single regression) and the CRIq*MRIq interaction; in Model 3 sleep duration was added (R^2^ = 0.040, *p* < 0.005 in the single regression); Model 4 added the sleep*CRIq interaction; Model 5 added the Med-diet score (R^2^ = 0.012, *p* = 0.125 in the single regression) and its interaction with CRIq; finally, Model 6 included all four predictors (CRIq, MRIq, sleep, Med-diet) and their interactions. All the models were statistically significant. Model 5, which included the four predictors, explained the highest proportion of variance (47%), outperforming the previous model and showing the lowest AIC. In Model 5, CRIq, MRIq and Med-diet emerged as significant predictors whereas sleep and its interaction with CRIq were not, despite sleep being a significant predictor in Model 3. In Model 5, the interaction MRIq*CRIq was significant (Fig. 2 panel A), as well as the Med-diet*CRIq interaction (Fig. 2 panel B).

#### Study of long-term memory

The above described methodological approach was adopted also for long-term memory.

The regression analysis of the variable ‘number of positive lifestyle factors’ on long-term memory showed that the effect was significant, but modest and the model explained 13% of the variance (see Table 4). Each additional healthy behavior was associated with a 0.29-point increase in memory scores, although no clear linear trend emerged (Supplementary Fig. 2). Participants who adopted all four healthy behaviors reached an expected long-term memory score of 0.25, corresponding to a performance around 51% of the sample.

Secondly, the hierarchical regression approach was applied to the long-term memory domain (see Table 6 for more details). Model 1 included the CRIq as predictor (R^2^ = 0.101, *p* < 0.001 in the single regression); Model 2 added MRIq (R^2^ = 0.027, *p* < 0.005 in the single regression) and the CRIq*MRIq interaction; Model 3 added sleep (R^2^ = 0.023, *p* < 0.005 in the single regression); Model 4 added the sleep*CRIq interaction; Model 5 added Med-diet (R^2^ = 0.0035, *p* = 0.39 in the single regression) and the Med Diet*CRIq interaction; Model 6 included all four predictors and their interactions.

**Table 6:** Results of the hierarchical regression analyses on the Long-term Memory scores extracted from PCA.

	**R**^**2**^	**Adjusted R**^**2**^	**AIC**	***F***	***p***	**Beta**	**t**	***p***
MODEL 1	.15	.11	568	3.45 (10,193)	< .001			
CRIq						.34	5.01	< .001
MODEL 2	.16	.11	569	3.13 (12,191)	< .001			
CRIq						.32	2.4	.017
MRIq						.07	-0.88	.381
MRIq*CRIq						.08	1.1	.273
**MODEL 3**	**.18**	**.12**	**567**	**3.22 (13,190)**	** < .001**			
CRIq						.32	2.29	**.023**
MRIq						.05	-1.08	.283
MRIq*CRIq						.09	1.27	.204
sleep						.13	1.93	**.056**
MODEL 4	.18	.12	568	3.05 (14,189)	< .001			
CRIq						.33	-.28	.778
MRIq						.06	-.92	.361
MRIq*CRIq						.08	1.11	.268
sleep						.14	-.63	.53
sleep*CRIq						.06	.96	.34
MODEL 5	.20	.13	568	2.95 (16,187)	< .001			
CRIq						.33	1.11	.27
MRIq						.05	-.91	.365
MRIq*CRIq						.08	1.08	.282
sleep						.15	-.65	.516
sleep*CRIq						.07	.99	.326
Med-diet						-.01	1.87	.063
Med-diet*CRIq						-.14	-1.96	.052
MODEL 6	.20	.13	570	2.76 (17,186)	< .001			
CRIq						.33	.1	.32
MRIq						.05	-.91	.365
MRIq*CRIq						.07	.95	.341
sleep						.15	-.61	.541
sleep*CRIq						.07	.9	.369
Med-diet						-.004	1.84	.068
Med-diet*CRIq						-.14	-1.84	.067
Med-diet*CRIq*MRIq*sleep						.04	.07	.946

CRIq, Cognitive Reserve Index questionnaire; MRIq, Motor Reserve Index questionnaire; Med-diet, Mediterranean Diet questionnaire

All models were statistically significant. Model 3, which included CRIq, MRIq and sleep, had the greater predictive power compared to Model 2 and showed the lowest AIC. In Model 3, the CRIq and sleep were significant, while neither the MRIq, nor its interaction with CRIq was significant.

## Discussion

This study aimed to investigate the role of four lifestyle factors -cognitive reserve, lifelong engagement in physical activity, dietary patterns, and sleep duration- on cognition in healthy aging, adopting a data-driven approach. In a sample of 204 healthy older adults, these factors showed different patterns of association depending on the cognitive domain considered: the association was stronger for executive functions, whereas it was more limited, although significant, for episodic long-term memory. Adopting all four positive lifestyle behaviours was linked to executive functioning levels exceeding those of 65% of the sample, while long-term memory performance exceeded that of 51%.

Among the four lifestyle factors, cognitive reserve accounted for the largest proportion of variance, explaining 42% of the variance in executive functions (Model 1) and 15% in long-term memory (Model 1). It was followed by physical activity and dietary habits, whereas sleep showed weaker associations (see Table 5 and Table 6). In a series of hierarchical regression models, MRIq*CRIq and Med-diet*CRIq interactions positively influenced EF, but not long-term memory.

Regarding the difference between executive functions and long-term memory, our results are in line with the study by Ngandu et al. [35] which involved 1′200 healthy participants (aged 60–77) and employed a two-year multidomain intervention including diet, exercise, and cognitive training: effects of these lifestyle factors were significant on executive functions (Cohen’s* d* = 0.129), but not on memory (Cohen’s* d* = 0.052). Taken together, these results suggest that executive functions may be more sensitive and responsive to environmental influences than episodic long-term memory. These environmental influences include specific forms of cognitive training capable of challenging and hence boosting executive functioning. In line with this interpretation, two recent reviews [66] [67] indicate that training on episodic memory typically shows more modest and less generalizable effects, whereas training on executive functions is more often associated with broader and more robust cognitive gains. It is possible that the frontal lobe, specifically the prefrontal cortex, retains some capacity to maintain and leverage the circuits necessary for carrying out complex functions, consistent with the compensatory scaffolding theory [68, 69]. These findings contribute to a finer-grained understanding of the cognitive benefits of a healthy lifestyle by highlighting domain-specific effects that may remain undetected if one considers global cognitive functioning [14, 26].

Cognitive reserve, the most extensively studied lifestyle factor, refers to the brain’s capacity to maintain cognitive functioning in aging by efficiently adapting and reorganizing neural resources in response to challenges or damage. Remaining cognitively active across the lifespan has profound protective effects on long-term cognition, with higher levels of cognitive reserve being associated with reduced cognitive decline and a lower risk of dementia [4, 70, 71]. Education in early life plays a crucial role for future cognition and dementia risk [17], as do occupation [72, 73] and lifelong cognitive engagement [74]. In fact, mental activity during both midlife and later years is associated with better cognitive performance and a lower risk of cognitive decline and dementia [75].

Different mechanisms may underlie these neuroprotective effects. Regular cognitive engagement contributes to increased neuroplasticity, including a higher number of synapses and higher BDNF levels, which increase not only after physical activity but also following cognitive activity [15, 75, 76].

When examining lifestyle factors across cognitive domains, interesting interactions are observed for EF. Indeed, EF are predicted by CRIq, physical activity and dietary habits, and by their interaction. Specifically, the MRIq*CRIq and Med-diet*CRIq interactions suggest that the association between exercise and EF is stronger when cognitive reserve is low, while it becomes negligible when cognitive reserve is high (Fig. 2A). Similarly, when the diet is unhealthy, the difference between high and low CRIq is substantial; when the diet is healthy, the difference between high and low CRIq is reduced (Fig. 2B). These findings show that motor activity and dietary habits are significantly associated with executive functions, particularly in individuals with low cognitive reserve. In both cases, and especially for diet, the effect appears to be more compensatory than enhancing, meaning that even when cognitive reserve is low, EF decline may be contrasted by positive habits such as physical activity and a healthy diet.

A similar pattern of results was reported by Chan et al. [77], where participants with high and low cognitive reserve showed different effects of total grey matter volume on performance in Cattell’s test. Specifically, total grey matter volume predicted cognitive performance only in the low cognitive reserve group, whereas no significant association was found in the high cognitive reserve group. This suggests that cognitive reserve is such a powerful modifier of cognitive functioning that overall performance cannot be fully explained by a generic volumetric index.

Regarding lifestyle factors, the literature does not establish a clear hierarchy of relative importance. For this reason, we relied on a data-driven approach based on R^2^ indices which highlighted cognitive reserve as the strongest contributor, followed by physical activity, sleep, and diet. When these factors were entered into hierarchical regression models, we observed that the contribution of motor activity and dietary habits was relatively limited. This is consistent with findings from the FINGER study [35], suggesting that interventions may have a positive impact on executive functions and processing speed i.e., more fluid cognitive functions, but their main effect may be to maximize existing cognitive resources rather than produce substantial gains [78].

Diet, assessed through the Med-diet questionnaire, also showed significant associations with EF. This finding is consistent with several studies demonstrating a positive relationship between adherence to the Mediterranean diet and cognitive performance [79, 80], and in particular with the study by Fostinelli et al. [63], in which a high Med-diet nutritional score was associated with better executive function in a group of healthy older adults from Northern Italy. In fact, a healthy diet, rich in vegetables, fruit, legumes, fish, olive oil and other components, has multiple benefits, including antioxidant, anti-inflammatory, and neuroprotective effects [81]. However, it remains unclear which cognitive functions benefit most from the Mediterranean diet, given the heterogeneity and conflicting results reported in meta-analyses [79, 82].

Finally, regarding the sleep factor, it was significant for EF when considered individually (Model 3), but lost significance when the sleep*CRIq interaction was included (Model 5). This lack of significance may be due to several reasons: in this study, sleep was assessed through a self-reported estimate, which may be biased, as individuals tend to over- or under-estimate how long they sleep. Moreover, sleep quality may be even more important than sleep duration when aspects such as feeling rested, sleep efficiency (the proportion of time spent asleep between falling asleep and waking up), time spent in different sleep stages, and sleep fragmentation are considered [34, 83].

Interestingly, in our study we did not focus only on variables that reflect a person’s current status (such as sleep duration, dietary habits over the past year, or total grey matter volume), but also on life-long habits, such as the motor activity throughout the entire adult lifespan as measured by section IV of the MRIq [42]. Our results suggest that the scaffolding of EF is built not only in the present, but progressively over time through the continued adoption of positive lifestyle behaviors. Beyond the contribution of cognitive reserve, indeed, the CRI*MRIq and the CRI*MedDiet interaction effects appear to be comparable (see the Beta values in Table 5).

From a methodological perspective, additive and moderation models were adopted to investigate the role of individual lifestyle factors in cognitive aging. Additive models allow the estimation of the independent and cumulative contribution of each factor (e.g. [26, 37]). Accordingly, a composite variable reflecting the number of positive lifestyle factors was created and included in separate regression analyses on executive functions and long-term memory. This type of analysis allowed us to quantify the cognitive benefit: individuals adopting all four positive lifestyle behaviors performed better than 65% of the sample in executive functioning, whereas for long-term memory the advantage was more modest, corresponding to performance levels above approximately 50% of the sample.

By contrast, additive models do not capture the relative importance of each factor nor their interactions. Since cognitive functioning is a complex, dynamic and interactive system, moderation models (e.g. [14]) were used to examine whether the association between lifestyle factors and cognition varies as a function of other variables, such as cognitive reserve.

In conclusion, this study offers a novel contribution in that it examines key protective factors across different cognitive domains, while controlling for relevant covariates (sex, smoking, alcohol consumption, and number of chronic conditions) and quantifies their contribution to cognitive performance: individuals who adopt all lifestyle factors show executive function scores higher than 65% of the sample average. This reflects a typical real-world scenario, in which most older adults present a combination of both protective and risk factors. Among all predictors, cognitive reserve explained the largest proportion of variance, followed by physical activity, diet, and sleep duration.

### Future perspectives and limitations

Future research should adopt longitudinal designs to assess the long-term impact of the observed effects. It would also be valuable to investigate additional protective factors, such as bilingualism, which has been associated with advantages in executive functioning and language processing in older adults [84].

Limitations of the present study include its cross-sectional design, which does not allow for causal inferences. Many lifestyle measures relied on self-reported data, which may be subject to social desirability bias or under-/overestimation. Third, the sample was relatively well-educated, as indicated by the CRIq scores. This may be partly explained by the fact that the study required participation via an online video call, which many individuals managed independently. However, this methodological choice enabled the inclusion of elderly participants from different regions across Italy, enhancing the geographical representativeness of the sample. An additional limitation concerns the fixed order in which the neuropsychological tests were administered, which may have induced fatigue effects in the final tests and potentially influenced participants’ cognitive performance. To partially mitigate this potential fatigue effect, the final two measures consisted of self-report questionnaires (BIS-15 and HADS), which are less cognitively demanding than performance-based tasks.

## Supplementary Information

Below is the link to the electronic supplementary material.

Supplementary file3

## Data Availability

Data can be found at the open science framework (https://osf.io/yd9kq).
