# Supplementary material for: Protecting the aging mind: how cognitive reserve and lifestyle factors relate to executive functions and long-term memory
Source: Aging Clin Exp Res. 2026 Mar 26;38(1):118. doi: 10.1007/s40520-026-03362-y (PMC13139212; doi:10.1007/s40520-026-03362-y)

**
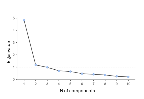
Supplementary Table 1:** Neuropsychological tests administration order.

Abbreviations: Mini-Mental State Examination (MMSE); Free and Cued Selective Reminding Test- Immediate Free Recall (FCRST- IFR); Free and Cued Selective Reminding Test- Delayed Free Recall (FCRST-DFR); Alternation Subtest of the Edinburgh Cognitive and Behavioral ALS Screen (ECAS 12).

The table reports the order of tests administration; the overall assessment protocol began with the Cognitive Reserve Index questionnaire (CRIq) and Motor Reserve Index questionnaire (MRIq) and concluded with the Barratt Impulsiveness Scale (BIS-15) and the Hospital Anxiety and Depression Scale (HADS).

| **Neuropsychological tests administration order** |
| --- |
| MMSE |
| Digit Span forward |
| Digit Span backward |
| Free and Cued (FCSRT) -IFR |
| Raven's Progressive Matrices |
| Stroop Test |
| Free and Cued (FCSRT) -DFR |
| Phonemic Fluency |
| Semantic Fluency |
| Alternate Fluency |
| ECAS 12 |

**Supplementary Figure 1.** Scree-plot used to determine the number of components to retain in the Principal Components Analysis.


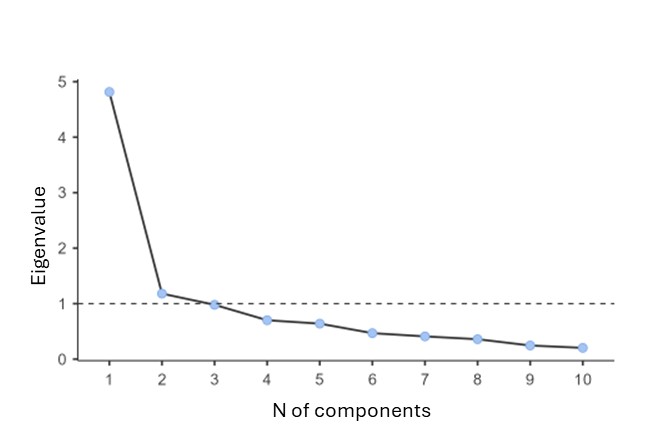


**Supplementary Figure 2.** Relationship between the number of lifestyle factors adopted (namely CRIq, MRIq, sleep, and Mediterranean diet) and long-term memory performance. The overall effect is small but statistically significant (see Table 4). Each additional healthy behavior is associated with a 0.29-point increase in long-term memory, although no clear linear trend emerges. Those who adopt all four factors reach an expected score of 0.25, corresponding to a performance around 51% of the sample.


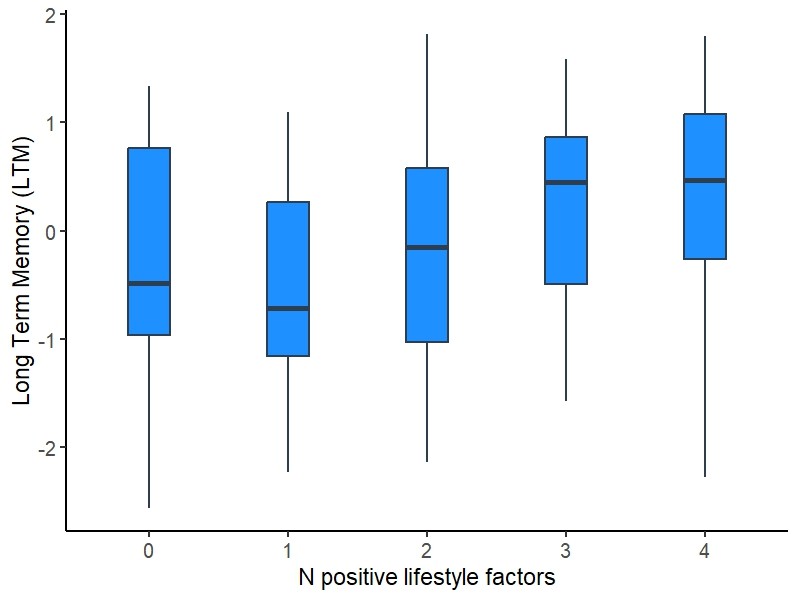

Supplement: Supplementary file 1 — Supplementary file3 [file 40520_2026_3362_MOESM1_ESM.docx]
